# Exploring Breakthroughs in Three Traits Belonging to Seed Life

**DOI:** 10.3390/plants11040490

**Published:** 2022-02-11

**Authors:** Angel J. Matilla

**Affiliations:** Departamento de Biología Funcional (Área Fisiología Vegetal), Facultad de Farmacia, Universidad de Santiago de Compostela, 15782 Santiago de Compostela, Spain; angeljesus.matilla@usc.es; Tel.: +34-981-563-100

**Keywords:** preharvest sprouting, MKK3, maternal and paternal expressed genes, imprinted genes, polycomb repressive complex 2, mRNA processing bodies, ribonucleic binding proteins, monosomes

## Abstract

Based on prior knowledge and with the support of new methodology, solid progress in the understanding of seed life has taken place over the few last years. This update reflects recent advances in three key traits of seed life (i.e., preharvest sprouting, genomic imprinting, and stored-mRNA). The first breakthrough refers to cloning of the mitogen-activated protein kinase-kinase 3 (MKK3) gene in barley and wheat. MKK3, in cooperation with ABA signaling, controls seed dormancy. This advance has been determinant in producing improved varieties that are resistant to preharvest sprouting. The second advance concerns to uniparental gene expression (i.e., imprinting). Genomic imprinting primarily occurs in the endosperm. Although great advances have taken place in the last decade, there is still a long way to go to complete the puzzle regarding the role of genomic imprinting in seed development. This trait is probably one of the most important epigenetic facets of developing endosperm. An example of imprinting regulation is polycomb repressive complex 2 (PRC2). The mechanism of PRC2 recruitment to target endosperm with specific genes is, at present, robustly studied. Further progress in the knowledge of recruitment of PRC2 epigenetic machinery is considered in this review. The third breakthrough referred to in this update involves stored mRNA. The role of the population of this mRNA in germination is far from known. Its relations to seed aging, processing bodies (P bodies), and RNA binding proteins (RBPs), and how the stored mRNA is targeted to monosomes, are aspects considered here. Perhaps this third trait is the one that will require greater experimental dedication in the future. In order to make progress, herein are included some questions that are needed to be answered.

## 1. Starting: Key Biological Traits about Seed Dormancy and Germination Mechanisms

The seed stage is a key life-cycle stage for many plants. Higher plants use the seeds for their perpetuation through successive generations. Mature seeds are highly resistant entities favoring plant dispersal. Seeds constitute also the basis of agriculture, and genes affecting seed dormancy and germination are among those under the strongest selection. The switch-off of translation between seed maturation and seed germination makes seeds a unique system to study developmentally regulated translation. Accordingly, seed germination is the first critical step of the plant life cycle and the foundation of agricultural production. The decision to germinate gravitates in a complex network of developmental and environmental signals to ensure seedling survival. The in-depth study of the seed’s life is being carried out under different scientific approaches, justified by the following molecular and genetic findings: (i) the endosperm (i.e., triploid nutritive support tissue with a 2:1 maternal-to-paternal genome ratio) is essential to repress dormant seed germination by releasing abscisic acid (ABA), which blocks embryonic growth. Although the interaction between endosperm–embryo is largely elusive, the flow of nutrients from mother to embryo is essentially unidirectional during the whole process of seed-life. Thus, early seedling growth is supported by catabolism of stored reserves of protein, oil, or starch accumulated in stored tissues during seed maturation [1,2,3,4]. (ii) Germination on the mother plant (preharvest sprouting, PHS), can also occur in crops, which is an agronomically and industrially undesired trait that compromises yield, nutritional and processing quality. Annual losses due to PHS are likely to approach $1 billion US dollars worldwide. Orthodox seeds acquire dormancy by thus avoiding PHS [5,6,7]. (iii) Orthodox and viable dry seeds are alive because they have acquired desiccation resistance, a feature accomplished together with longevity (storability) at the beginning of maturation [8,9,10,11]. (iv) Interestingly, dry and viable seeds store a multitude of transcripts to be used at the beginning of the germination process [12,13]. (v) Reactive oxygen species (ROS) and nitric oxide (NO) play fundamental roles in seed-life [14,15,16,17]. (vi) All the above vital events that occur in seeds are coordinated by transcription factors (TFs) such as ABI3, ABI4, and ABI5 [18,19,20,21,22,23], and phytohormones, being the regulatory mechanisms underlying abscisic acid (ABA) and gibberellins (GAs) crosstalk, intensively documented during seed dormancy and germination. However, identification of the ABA and GAs synthesis/degradation pathways, their feedbacks, and their impact on the regulation of dormancy and germination is far from clarified [23,24,25,26]. On the other hand, the involvement of ethylene in the regulation of seed dormancy and germination was recently updated and discussed [27,28,29,30]; and (vii) the life of this propagule is strongly influenced by external signals (e.g., light, nitrate, humidity, temperature) [31,32,33,34,35]. Taken together, all the events previously referred occur, among other instances, in human-used seeds. That is, in agronomically important crops that are therefore related to the world economy. Finally, the seeds of several species are used as biological systems in order to advance knowledge of the complex puzzle that constitutes the seed life and perpetuation of these entities that have been key in the colonization of dry land [36]. This update summarizes recent breakthroughs in our knowledge of some aspects highly involved in the life of orthodox seeds.

## 2. Preharvest Sprouting: Recent Progress and Economical Repercussions

Seed dormancy, a key quantitative trait for the prevention of PHS, has essential repercussion in plant survival and crop production. PHS is a complex trait influenced by genetic and environmental factors [37,38,39,40]. However, the intensity of this trait decreases during plant domestication to ensure that crops successfully germinate in the field. This is true in the case of wild and cultivated barley (*Hordeum vulgare*) [41]. If the rain comes before harvest, the mature seeds can germinate (i.e., PHS or vivipary) on the mother plant, causing substantial damages and crop losses. Considering that PHS broadly constitutes the opposite process to primary dormancy, at an experimental level, the natural phenomenon of PHS can be used to collaborate in the study the dormancy mechanisms. On the other hand, it seems logical to understand that achieving tolerance to PHS (i.e., breeding PHS-resistant varieties) is one of the goals of actual seed research. Consequently, it is not surprising that the focus of PHS research in recent years has been on identifying individual genes impacting seed dormancy. *Osaba1* was the first PHS-related mutant to be identified in rice [42]. This mutant harbors a point mutation in the zeaxanthin epoxidase gene, is strongly viviparous with wilty phenotype, and displays a low ABA level with almost no further increase in its levels upon drought.

PHS is a trait controlled by multiple QTLs [39]. In rice, an important cereal crop, more than 165 QTLs associated with seed dormancy or PHS resistance and located on different chromosomes have been identified. Similar to rice, several research groups identified causal genes for the major dormancy QTL in other cereals such as barley and wheat (*Triticum aestivum*). Dormancy QTLs have been found on all chromosomes in both crops [43]. However, although PHS-associated QTL or genes have been reported and cloned, the PHS molecular mechanism remains little-known. Since two major dormancy QTLs (*SD1* and *SD2*) have been detected in barley grains at the beginning of the present century, the PHS process has been extensively investigated and new advances are constantly emerging. In the past few years, it was eventually revealed that alanine aminotransferase (*AlaAT*) is the causal gene for the major grain dormancy QTL *Qsd1* (*SD1*) in barley [44], and that mitogen-activated protein kinase–kinase 3 (*MKK3*) is the causal gene for the major grain dormancy QTLs *Qsd2-AK* (*SD2*) in barley and *Phs1* in wheat [41,45]. *MKK3* was found to control seed dormancy in wheat and barley using a map-based cloning method [41,45]. *Phs1* in wheat is an ortholog of *SD2* in barley [45]. *Qsd2-AK* at *SD2* acts as a single major determinant explaining the difference in seed dormancy between the dormant cultivar “Azumamugi” (Az) and the nondormant cultivar “Kanto Nakate Gold” (KNG) [41]. QTL *Qsd2-AK* (*SD2*) is a component of the MAPK cascade (i.e., MKKK, MKK, and MAPK) which is evolutionarily conserved in photosynthetic eukaryotes, including Arabidopsis [41]. In summary, Nakamura’s group provided for the first time the key to finding plants refractory to PHS. Therefore, cloning of the *MKK3* wheat gene (*TaMKK3*) has been essential to produce improved varieties that are resistant to PHS. Thus, transformation with the *TaMKK3* susceptible allele caused a large increase in PHS susceptibility in dormant backgrounds [41]. The N(asparragine) 260 T(threonine) mutation reduces TaMKK3 kinase activity [41]. Accordingly, the N260T-substituted dormant *MKK3* recessive allele cannot efficiently transmit phosphorylation signals in the MAPK module for germination, delaying germination and conferring the hyperdormant phenotype. Introducing the N260T mutation into all of the functional wheat homologs of MKK3 will be a novel way to increase seed dormancy in wheat. On the other hand, a more recent update provides unique insight into the genetic mechanisms governing PHS in bread wheat [46]. It is important to emphasize that these recent advances can be directly applied in breeding programs to improve PHS tolerance in studied species. Thus, causal genes are now available for marker-assisted selection to improve PHS tolerance in barley and wheat [47].

Together with other map kinases, MKK3 also works in ABA signaling and seed dormancy in Arabidopsis, and loss of *AtMKK3* function has led to ABA-hypersensitive seed germination [48,49,50]. In 2019, a solid advance occurred in seeds of rice. Thus, the overexpression of *MKKK62* in the embryo and endosperm significantly decreased seed dormancy levels, while the overexpression in the testa and husk did not. *MKKK62*-overexpressing rice lines lost seed dormancy, increased PHS and ABA sensitivity was decreased. High levels of MKKK62-mRNAs were found at the late stage of rice-seed maturation, suggesting that *MKKK62* affected seed traits. MKKK62 (rice has 75 *MKKK*s) interacted with MKK3, while MKK3 interacted with MAPK7 and MAPK14 (from in yeast two-hybrid experiments) [51]. Knock-out experiments confirmed that *MKKK62*, *MKK3*, *MAPK7,* and *MAPK14* (i.e., the entire rice MAPK module) were involved in the regulation of seed dormancy in rice. In other words, protein modification by phosphorylation plays a key role in controlling rice seed dormancy. Interestingly, the results from [51] also indicated that regulation of seed dormancy by *MKKK62* is a common phenomenon among cereals. On the other hand, at least five MKKKs (AtMKKK14/15/16/17/18) are able to activate AtMKK3 in Arabidopsis; but none of these five genes affect seed dormancy when they are overexpressed [52]. The results from [51] also prove that seed dormancy in rice is negatively regulated by *MKKK62* and insinuate that the regulation of seed dormancy distinguish between *Arabidopsis* and rice. Very recently, the ENHANCED RESPONSE TO ABA8 (ERA8) mutant increases seed dormancy and, consequently, PHS tolerance in the soft white wheat ‘Zak’ [53]. This work suggests that the ERA8 phenotype results from the MKK3-A-G1093A mutation and for that reason, is being used to introgress ERA8 into soft white winter wheat to improve PHS tolerance by increasing ABA sensitivity. An in-depth study of the relationship between MKK3 and ABA will produce a great scientific step not only in breeding programs, but also in model systems such as Arabidopsis or *Medicago truncatula*. Referring to this, two genes in the Raf subfamily of MKKK genes, *Raf10* and *Raf11,* were found to positively regulate seed dormancy in *Arabidopsis* [54]. The functions of these genes are still unknown in crop species like rice. But an influence on seed dormancy should not be ruled out.

Apart from the study of QTLs involved in seed dormancy, other approaches are currently underway for understanding of molecular mechanisms underlying PSH. There is a lot of evidence indicating that ABA and sugars participate in the regulation of seed dormancy and germination [55,56]. Data presented in [57] strongly support that the sugar accumulation is the essential cause of PHS in rice *phs8* T-DNA insertion mutant and the ABA level is lower in the mutant than in the wild type. The alterations in the content of free monosaccharides and oligosaccharides were more notable in the endosperm of *phs8*. It is of interest to note that the *OsABI3* and *OsABI5* transcripts decreased in mutant seeds, and that overexpression of *OsABI3* and *OsABI5* could partially rescue PHS in the *phs8* mutant. Interestingly, both glucose and sucrose suppressed the expression of *ABI3* and *ABI5*, suggesting that sugar is sufficient to suppress the ABA signaling pathway (see Figure 6 from [57]). Curiously, PHS8 is located in seed-dormancy-related QTLs, which leads us to suppose that any natural variation of *PHS8* may affect seed dormancy. More recently, another T-DNA insertion dominant mutant (*phs9-D*) was characterized in rice. The authors conclude that PHS9 plays an important role in PHS regulation through the integration of ROS and ABA signalings [58]. However, the role of ROS in PHS control requires much more investigation [16]. On the other hand, it is well-known that the expression of *ABI5*, the ortholog of *VP1*, is regulated by *ABI3*, expression of which is required for appropriate *ABI5* expression. ABI3 and ABI5 control the seed sensitivity to ABA [23]. ABI5-binding protein (AFP) induces ABI5 degradation [24,59]. In order to understand the mechanisms underlying seed dormancy or PHS tolerance in common wheat (*Triticum aestivum*), the possible role of TaAFP in seed dormancy was developed, concluding that TaAFP is a negative regulator in seed dormancy [60]. Likewise, this magnificent work concludes that TaAFP-B had a 4-bp InDel in the 5′UTR, which affected the mRNA stability, mRNA transcription expression level, and GUS activity and was significantly associated with PHS tolerance. Finally, QTL-seq analysis was recently performed to identify for the first time QTLs associated with PHS in cucumber (*Cucumis sativus*) using an F2-segregating population, and two QTLs were detected and two candidate genes (*Csa4G622760* and *Csa4G622800*) were proposed [61]. Taking into account the remarkable research on PHS carried out in the last decade, we should be hopeful regarding the avoidance of the great losses produced by PHS in cereal crops. Although a number of QTL or genes related to PHS have been reported in cereals, the molecular mechanism underlying PHS remains largely elusive.

## 3. Endosperm–Embryo Relationships: Imprinted Gene Expression

In angiosperms, reproduction occurs by double fertilization whereby one of the two sperm cells in the pollen grain fertilizes the egg cell to produce the embryo; while the other one fertilizes the binucleate central cell to generate the triploid (3n) endosperm, which nourishes the embryo. Although with very few exceptions, the endosperm and embryo have essentially the same genotype and markedly different developmental programs. However, the endosperm has two maternal doses of the genome, whereas the embryo has one paternal dose [1,62]. The endosperm does not contribute with genetic material to the next generation, but controls a good number of key processes for the development of the embryo (e.g., providing genetic and molecular signals [63]) and it is an important site of genomic imprinting in higher plants [64].

Genomic imprinting (i.e., uniparental gene expression) is an epigenetic phenomenon in higher plants whereby genetically identical alleles (i.e., parental alleles) have unequal expression depending upon their parental origin [65,66]. The first imprinted gene was discovered in the maize endosperm 51 years ago by phenotypic identification [67]. Maize is also among the several plant species in which gene imprinting has been studied most comprehensively in recent years [68,69]. *Arabidopsis thaliana* has been reported to display genomic imprinting on at least 436 genes in its seed endosperm. In mammals, approximately 80% of the imprinted genes are clustered on chromosomes; but in plants, the majority of the imprinted genes are scattered on chromosomes. At present, and unlike in mammals, a wide debate exists regarding the conservation of the imprinted status in plants (for update see [70]). That is, in developing the seeds of higher plants, some genes show biased gene expression of the allele descended from a particular parent [71]. For this reason, the imprinted genes are classified into maternal expressed genes (*MEG*s) and paternal expressed genes (*PEG*s). Probably *PEG* and *MEG* are subject to different selective pressures. In mammals, approximately 80% of the imprinted genes are clustered on chromosomes, and in plants most of the imprinted genes are scattered on chromosomes. Recently, *PEG* and *MEG* genes identified under positive selection are involved in processes such as auxin biosynthesis (e.g., *YUCCA10*, *TAR1*) [72]. On the other hand, the number of *PEG*s was much lower than the number of *MEG*s in species as *A. thaliana*, maize, *B. napus*, sorghum and *A. lyrata*. The unbalanced *MEG*s and *PEG*s in plants agreed with the maternal-offspring co-adaptation theory, indicating that the maternal genes were more favored during natural selection [70]. In the present year, a work on the genomic imprinted genes of dicot *B. napus* endosperm provided 297 imprinted genes, including 283 *MEG*s and 14 *PEG*s. More specifically, (i) 36 of 297 imprinted genes were continuously imprinted during endosperm development; (ii) only 26 imprinted genes were specifically expressed in endosperm, while other genes were also expressed in other tissues of *B. napus*, rather than specifically in endosperm; and (iii) a total of 109 imprinted genes were clustered on rapeseed chromosomes [73]. Recently, imprinting status in two closely related dicot species, Arabidopsis and *Capsella rubella*, was analyzed, revealing that less than one-third of orthologous genes are imprinted in both species, and that genomic imprinting is a highly dynamic process [74]. On the other hand, in dicot *Ricinus communis* were identified 209 genes in reciprocal endosperms with potential parent-of-origin specific expression, including 200 and 9 maternally and paternally expressed genes, respectively. More information on *MEG*s and *PEG*s in mono- and dicot species can be reviewed [1]. Likewise, increasingly imprinted genes have been identified and characterized in plant species (for reviews see [64,75]). Although many imprinted genes have been identified in plants, the functional significance of the majority of them remains unknown both in seed life and in other plant organs, which constitutes a strong weakness of this remarkable breakthrough in development biology. Functional studies are necessary to unravel relevant imprinted characterized genes (reviewed in [76]). However, a positive aspect of the study of the imprinting phenomenon is the conclusion that the genes subjected to imprinting are largely conserved across kingdoms [65,77,78]. In contrast to *B. rapa*, genomic imprinting in diploid (*Aegilops* spp.), tatraploid, and hexaploid wheat (*Triticum* spp.), showed evolutionary conserved nature of imprinting status during polyploidization [78].

During the present century, it was evidenced that epigenetic modifications play notable functions in developing seeds [79]. Interestingly, the endosperm has a significant level of hypomethylated DNA in maternal alleles and a looser chromatin structure. As in plant genome methylation, Arabidopsis and rice endosperm is hypomethylated at short transposable elements and related sequences that reside near genes [76,80]. *A. thaliana* has been reported to display genomic imprinting on at least 436 genes in its seed endosperm. Maize, sorghum, and rice endosperm DNA are also hypomethylated [81,82]. Conversely, the embryos are hypermethylated [81]. DNA methylation is essential for the repression of transposable elements and the regulation of gene expression. At present, there is no direct evidence for how dynamic DNA methylation and differentially methylated genes regulate seed development. Curiously, the expression of the imprinted genes was not tightly linked to DNA methylation in castor bean [83]. It is noteworthy that imprinted genes expressed in the endosperm of *A. thaliana* are rapidly evolving due to positive selection; such positive selection is preferentially associated with imprinted paternally expressed genes [72].

DNA methylation is a crucial epigenetic modification involved in many biological processes, including genomic imprinting [84]. The imprinting process includes trimethylation of histone (i.e., H3K27me3) and DNA methylation without altering the genetic sequence [69,85]. In angiosperms, H3K27me3 predominantly marks the maternally imprinted alleles of paternally expressed genes, whereas DNA methylation predominantly marks paternally imprinted alleles of maternally expressed gene [76]. As summary, in mammals and higher plants imprinted genes, likely established prior to fertilization, are silenced through cytosine methylation, histone modifications or both. However, in mammals the epigenetic modifications at imprinted loci are deleted and re-established in each generation (i.e., imprinted alleles are targeted for silencing), while in plants most of the evidence so far is that pre-existing methylation present on both alleles in the parent plant is specifically removed during gametogenesis from the allele destined to be active in endosperm [86]. In higher plants, DNA methylation is maintained by the maintenance methyltransferase enzyme (MET1 in Arabidopsis) and is essential for propagating methylation marks on imprinted genes. However, in mammals, de novo methylation is also required to place the marks, while in Arabidopsis there is no evidence so far that de novo methylation has a role in imprinting. On the other hand, the evolution of imprinting in animals and higher plants still has many gaps. The characterization of the dynamic DNA methylome will be of the great help [87]. Imprinting is an evolutionary puzzle, as it bears the costs of diploidization without its advantages, namely, protection from recessive mutations. A recent review helps to clarify the complexity of this epigenetic puzzle [88].

The reason for the importance of imprinting is that the chemical modification produced in the DNA, which is transmitted to the offspring, changes the gene expression or the function of the gene product. Interestingly, for complex traits (e.g., rice grain size), some QTLs may also exhibit imprinting effects (i.e., manifesting different genotypic values between reciprocal heterozygotes) and hence are termed imprinted QTLs (iQTLs) [65]. Genomic imprinting primarily occurs in the endosperm [76,89]. As a possibility, the endosperm growth is suitable when *PEG*s (presumably promoting growth) and *MEG*s (presumably repressing growth) are jointly expressed with appropriate dosages. That is: do imprinted genes interact? Although *PEG*s and *MEG*s may be physically and functionally linked, they are possibly regulated by different mechanisms [90]. Although the main functions of imprinting in the endosperm are not fully understood, experiments in *A. thaliana* have demonstrated that imprinting defects in the endosperm may cause seeds to abort [80,90]. Conversely, few embryo processes were linked to genomic imprinting [69,91,92]. Several lines of evidence indicate that the endosperm and embryo exist in distinct transcriptional and chromatin states immediately after fertilization [93]. Summarizing the information to date, endosperm plays an active role in promoting embryo development and its epigenetic regulation could have consequences for embryo developmental programs. Unfortunately, little is known about gene imprinting in dicotyledons.

Given the interest in imprinting in higher plants, numerous updates have been published recently [69,70,80,94,95,96]. By using: (i) the rapid development of modern technologies (e.g., CRISPR/Cas9, protein-DNA interactions by CUT&RUN, high-throughput transcriptome sequencing, and single-cell RNA sequencing, among others), together with yeast two-hybrid tests; and (ii) the F1 hybrid seeds derived from reciprocal crosses of plants with different ploidy levels to provide different dosages of the parental genomes, a direct genome-wide survey of imprinted genes at the transcription level has become possible. Furthermore, the studies of imprinting in diverse plant species, especially in several important crops, have intensified [75,97]. In biological terms, paternal-excess crosses strongly promote seed development and big seeds; whereas maternal-excess crosses dramatically inhibit endosperm growth and the production of small seeds. These features indicate that an adequate balance between maternally and paternally derived genomes is responsible for both embryo and endosperm development.

**Figure 1:**
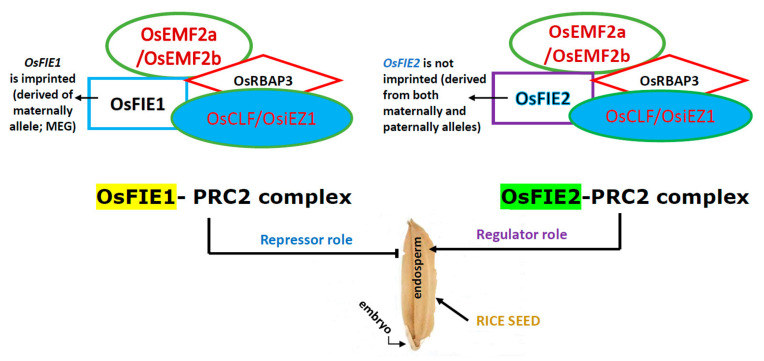
The role of polycomb repressive complex (PRC2) in rice endosperm. PRC2 is an evolutionarily conserved multimeric protein complex that has methyltransferase activity for Lys27 of histone H3 (H3K27), repressing gene expression. Mutation of the imprinted gene *OsEMF2a* induces autonomous endosperm development [108]. FIE: fertilization independent endosperm; EMF2: embryonic flower 2; RBPA3: WD-repeat protein; CLF and iEZ1: enhancer of zeste-like homologs. Adapted from [107,108].

Although the progress about genetical properties of imprinted genes is unquestionable (see above), aspects regarding the biological relevance of genomic imprinting still remain to be answered. The increased understanding of the role of PRC2 (polycomb repressive complex 2) in different plant species should be of great value to addressing many of the unanswered questions. PRC2, a subset of the PcG proteins, possesses an evolutionarily conserved epigenetic histone methyltransferase, is a major chromatin-modifying multi-subunity complex that catalyzes H3K27me3, and plays a role in safeguarding cellular identity [98], among other functions. H3K27me3 is a repressive epigenetic mark that results in compaction of chromatin, and is associated with genes with low expression levels and high tissue specificity (e.g., endosperm) (Figure 1). Plants lacking PRC2 components do not show severe embryonic phenotypes and most produce viable offspring [99]. The mechanism of recruitment of PRC2 to endosperm target genes is an intense and actual area of study. Therefore, how PRC2 epigenetic machinery is recruited to specific targets in plants remains largely unclear. However, some progress has occurred recently: (i) seed-coat initiation is controlled by an epigenetic regulator commonly used in developmental transitions [100]. This feature hints that recruitment of polycomb-group protein FERTILIZATION INDEPENDENT ENDOSPERM (FIE), acts as an epigenetic switch that may have been key to the evolution of the seed coat (revised in [1]); (ii) in lateral roots of *A. thaliana*, BASIC PENTACYSTEINS (BPCs) recruit PRC2 to the ABI4 (ABA INSENSITIVE 4) locus and repress ABI4 expression epigenetically by catalyzing the H3K27me3. That is, BPCs bind to the ABI4 promotor, repress ABI4 expression and physically interact with PRC2 [101]. It will be important to demonstrate all these facts in seed development. (iii) Interestingly, the PRC2 recruitment in Arabidopsis relies in large part on binding of trans-acting factors to cis-localized DNA sequence motifs [102]; and (iv) a short time ago, it was shown that SDG711-mediated H3K27me3 changed significantly in genes related to endosperm development, and that SDG711, a histone transmethylase, can directly bind to the gene body region of several starch-synthesis and amylase genes, respectively [103]. For more information on how PRC2 is involved in development and identification of its direct target genes, see [104].

In *Arabidopsis*, DNA methylation and some maternally expressed components of PRC2 are involved in the regulation of some imprinted loci in the endosperm [69]. PRC2 represses endosperm development both before and after fertilization. In other words, some components of PRC2 are maternally expressed and involved in the control of endosperm development [105]. However, the role of PRC2 in endosperm development in monocot is still unclear. Recent results provide strong evidence that rice PRC2 represses central cell proliferation and endosperm formation before fertilization [106,107]. PRC2 core components are broadly conserved and essential for the H3K27 trimethyltransferase activity of the complex [108]. Plants possess several different PRC2 complexes, such as FIS-PRC2 (FIS2), which is specifically involved in female gametophyte reproduction, and endosperm and seed development [109]. Endosperm development prior to fertilization is inhibited by FIS2, which acts in the female gametophyte and during endosperm development (for extension, see the update from [100]). On the other hand, the protein–protein interactions of each PRC2 component are important determinants of the activity of the PRC2 complex. Seeds inheriting maternal mutant alleles of these FIS-class genes abort due to a failure in endosperm cellularization and embryonic arrest, regardless of the paternal genotype. This maternal effect is observed because FIS2 is regulated by epigenetic genomic imprinting.

In conclusion, although great advances have taken place in this decade, there is still a long way to go to complete the puzzle regarding the role of genomic imprinting in seed development. Future investigations will refine the interplay between transcriptional, hormonal, signaling, and epigenetic controls operating in the regulation of endosperm growth. Thus, the interaction between PEGs and MEGs must be definitively confirmed in higher plants. Once it has been demonstrated that PRC1 and PRC2 directly interact, it is important to clarify the role of PRC1 in the functioning of PRC2 in the endosperm and demonstrate whether imprinting always preferentially affects the endosperm. Evolutionarily speaking, it is necessary to investigate if imprinting takes place in organisms other characterized [110,111,112]. Finally, it is worth knowing how PRC2 epigenetic machinery is recruited to specific targets in endosperm. This aspect is probably one of the most important epigenetic facets of developing endosperm.

## 4. The Stored mRNA: A Surprising Singularity in the Seed Life

Excepting dry, viable seeds, molecules of mRNA are generally short-lived. Different mRNAs transcribed during the last phase of development are stored in dry seeds of many species [113]. Seed-stored mRNA can survive for long periods. Some of these stored mRNAs are called ‘long-lived mRNAs’ because they remain active for a long time, the great majority being selectively translatable at the onset of germination [114], and the others are degraded. Although mRNA decay regulates mRNA levels, the regulation of stored-mRNA decay machinery remains still elusive in seeds. Dry rice and Arabidopsis seeds contain >17,000 and >12,000 stored mRNAs, respectively [115,116,117]. The seed-stored mRNAs encode many proteins of diverse physiological processes and cues [13]. However, not all of these mRNAs are considered ‘long-lived mRNAs’ because the temporality of translation is different between members of this population. In addition to mRNAs, dry seeds accumulate a large amount functional proteins involved in metabolism, transcription, and translation. Before the onset of desiccation and triggering of dormancy, embryos transcribe and store ‘long-lived mRNAs’ involved in the germination process (e.g., ABA catabolism-related enzymes, phospholipids, and calcium ion signaling-related proteins) [118]. The evidence that seeds translate stored mRNA during germination using stored ribosomes has been amply demonstrated [114]. However, ribosomes are inactive in dry seeds, and must be reactivated in order to germination process takes place. A task for ‘long-lived mRNAs’ once demonstrated that germination is not inhibited in the presence of transcription inhibitors; but it does when translational inhibitors were added to the medium [119,120]. One of the existing gaps is deciphering if the population of stored ‘long-lived mRNAs’ is sufficient to initiate germination. Anyway, the population of stored mRNA within dry seeds can sustain germination completion in several species [121]. The authors of [121] suggest that the classes of ‘long-lived mRNAs’ are highly conserved between dicot and monocot seeds. To clarify the distinct roles of proteins translated from long-lived mRNAs and de novo transcribed mRNAs, a proteome analysis was recently performed in germinating rice seeds. This leading work proposes that long-lived mRNAs support an initial energy production (e.g., glycolysis-related proteins) and activation of the translational machinery upon imbibition, whereas the de novo transcriptions accelerate the energy production after glycolysis [122].

So far, it has been unknown how fragmentation of seed-stored mRNA affects germination once rehydration occurred. However, there is no doubt that the cellular redox state affects RNA fragmentation by altering several key processes of seed life [16,123]. It seems also clear that the seed does not have mechanisms that resolve the mRNA fragmentation. Therefore, seed viability and aging are affected by fragmentation, and sometimes death occurs. Seed aging is a convoluted biological trait in which several interconnected molecular, biochemical, physiological, and metabolic events are involved. The seed-aging process is usually associated with the oxidation of macromolecules. Recently, Fleming et al. (2018) observed fragmentation of seed-stored mRNAs in soybean embryonic axis by transcriptomic analysis, and suggested that mRNA breakage occurs at random positions [124]. Later, Zhao et al. (2020) elegantly showed in Arabidopsis that the degradation of seed-stored mRNA is greatly correlated with seed-aging time. Stored mRNA of almost all the ubiquitination-related and heat-shock protein (HSP) genes analyzed decreased gradually in aged seeds. In addition, enzymes related to energy production and structural proteins of the cytoskeleton are also involved in this seed-stored mRNA degradation process [125]. Interestingly, the data from [125] also indicate that the majority of Arabidopsis seed-stored mRNA have a similar and constant rate of degradation (i.e., the time for the mRNA level to decrease by 50% is constant) during seed aging. These findings were ratified in canola and wheat, two valuable agricultural species [126]. This and previous work indicate that the damage or degradation of stored mRNA occurred randomly along the length of this RNA, and also propose that stored mRNA degradation during seed aging is a general phenomenon for seeds [124,125]. On the other hand, the degradation of ribosomal RNA is widely described during seed aging [127,128,129].

**Figure 2:**
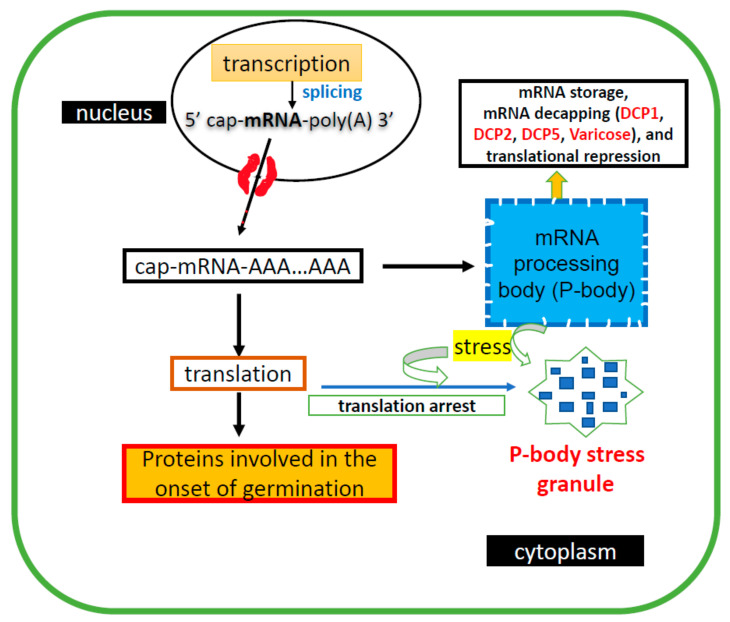
The stored mRNA, once in the cytoplasm, can be sent to P bodies located in the ER for processing; or, alternatively, included in the translation machinery. What controls this mRNA distribution is not known. Under stress, P bodies are agglutinated in granules and their translation is reduced. DCP (decapping enzyme), ER (endoplasmic reticulum).

Although many physiological properties of stored mRNA are known, the cellular conservation, stability, transcription, and translation of these molecules is not entirely clear yet [12,130]. Degradation of mRNA is a key process in the regulation of gene expression and elimination of defective mRNAs. The processing bodies (P bodies) are equipped with machinery for RNA degradation (Figure 2). These cytoplasmic bodies are RNA membraneless granules (i.e., liquid–liquid phase separation), constituted by ribonucleoproteins associated with both mRNA decay and translation repression. Plant P bodies share many protein components with yeast and mammalian P bodies and are conserved in eukaryotes. It is worth highlighting that P bodies possess targets of the mRNA decapping machinery (e.g., *ASL9*; [131]) and can also serve as translationally repressed mRNA reservoirs in regulating the homeostasis of mRNA translation [132,133,134]. Interestingly, the mRNA decay machinery directly targets *ASL9* transcripts for decay to balance cytokinin/auxin responses during developmental reprogramming [131]. In other words, P bodies are often considered to be the site for mRNA decay due to their content of decapping complexes (i.e., proteins related to mRNA decay), de-adenylation factors, 5′ to 3′ exoribonuclease, Argonaute 1 (key protein gene silencing), and factors involved in nonsense-mediated mRNA decay [135,136,137]. Therefore, it is not surprising that plants deficient in P-body components display severe developmental perturbations [132]. However, little is known about how decapping contributes to plant development. Recently, it was reported that mRNA decay is required to unlock cellular states during development [131]. On the other hand, mRNA may be sequestered in P bodies for degradation, or re-enter polysomal translation complexes, which may be related to the use of dry seed-stored mRNA during onset germination. However, this assumption still needs to be proved and generalized in angiosperms. Interestingly, P bodies are highly mobile and connected to actin filament and myosins via interaction with DECAPPING PROTEIN1 (DCP1) [138]. Decapping enzymes DCP1/2 present their genes in a single copy in the Arabidopsis genome, and together with VARICOSE (VCS) are part of the conserved components among eukaryotes’ decapping complex located in P bodies. The mRNA turnover carried out in this decapping complex is essential for postembryonic development in Arabidopsis. DCP2 form a complex catalyzing the removal of m7GDP caps from mRNAs [132,139]. Importantly, components of the cytoplasmic 5′-3′ mRNA decapping pathway (i.e., DCP1/2/5, VCS, PAT1 (which accumulates in P bodies) and exoribonuclease XRN4) [140] contribute to ABA signaling in Arabidopsis [141]. Other types of RNA granules together with P bodies may also be involved in controlling the translation in seeds [142].

On the other hand, P bodies can contain several RNA-binding proteins (RBPs), which stabilize and/or modulate the translation of target mRNAs for decay [136,143]. Importantly, binding of RBPs’ sequence elements in mRNA can both promote and repress translation [137]. RBPs are tightly bound RNAs that through one or multiple globular RNA-binding domains form ribonucleoprotein complexes that dynamically regulate the RNA’s fate and function. Thus, RBPs are versatile key players in the posttranscriptional control of mRNAs and candidates for regulating translation during seed germination [144,145,146]. However, few RBPs have been functionally explored in the seed world. Knowledge of the functional roles of RBPs in plants is lagging far behind regarding those in other organisms. Recent findings in the field have been collected and commented on [147]. Suggestively, some studies clearly indicate that many RBPs harboring RNA chaperone activity play essential roles in the regulation of RNA metabolism during plant growth and development (for more information, see [144]). Some RBPs as RZ-1A and glycine-rich (GR)-RBP (GRP1A), have been detected in rice seeds by proteomic analysis [148]. In 2013, it was demonstrated that RZ-1A and GRP1A are solid RBP candidates involved in seed desiccation while also preserving the stability of “long-lived mRNAs” [149]. In 2020, a solid experimental work was reported in *A. thaliana* leaves. This protocol developed the RNA interactome capture to identify proteins that interact with RNAs-poly(A) in living cells [150,151]. The use of this methodology in seeds will provide an outstanding breakthrough in the study of RBPs. In a recent and detailed work carried out in *A. thaliana*, the Bentsink group smartly identified 30 seed-specific RBPs and 22 dynamic RBPs from testa rupture to radicle protrusion period [152]. These authors argue, in a well-built discussion, the possible role of RBPs in the germination process. A major task in the seed world is to identify RNA targets and to understand how RBPs recognize substrate RNAs and how they interact with other protein factors to regulate posttranscriptional RNA metabolism during plant growth and development.

The Arabidopsis genome harbors hundreds of mRNA binding proteins, of which a large majority do not yet have an assigned function (see comments from Bai et al., 2017 [114]). Since it is known that monosomes are very abundant in Arabidopsis dry seeds, a complicated protocol was started to demonstrate if any protein of monosomes was bound to the seed-stored mRNA. Thus, Bai et al. (2020) proved that seed-stored mRNA can bind to monosome proteins (i.e., 50% of seed-stored mRNAs are bound to 80S ribosomes, mostly monosomes, whereas the other 50% are free-stored mRNA). In other words, transcripts that are associated with ribosome complexes in the dry state are translated (associated with polysomes) upon seed imbibition (see Tables 1 and 2 from [153]). On the other hand, a determined monosome population in dry seeds is enriched with proteins related to protection against oxidative stress. This feature suggests that specific mRNAs are preserved from oxidation until the translation machinery begins with the seed hydration. But the way to choose theses specific mRNAs is, at present, noteless. Chantarachot and Bailly-Serres (2018) suggested several possibilities for it [137]. Moreover, these authors also suggested that the conserved eukaryotic decapping VCS, a protein associated with monosomes and polysomes, can be involved in the degradation of seed stored-mRNAs not compromised in the germination process. However, how specific mRNAs are targeted to monosome complexes and are specifically translated during imbibition is at present unknown.

The complexity of the involvement of stored mRNA in the seed-germination machinery is considerable. Further analyses are required to identify and characterize which mechanisms are used by the seed to benefit from some of the mRNAs transcribed during the maturation period. The following questions, among others, will need to be answered in order to make progress. Thus:

(i) Is the population of stored-mRNA sufficient to initiate the germination process? Are the stored-transcripts encoding ribosomal proteins involved in the onset germination?
(ii) Since selective mRNA translation is a key feature of the seed germination process, which mRNAs are exclusively for that process, and how are they selected from among stored mRNAs?
(iii) Are the chosen mRNAs epigenetically marked?
(iv) Is the signaling network involved in germination compromised in the choice of mRNAs?
(v) What is the mechanism for the spatio-temporal choice of each transcript during maturation and onset germination? That is, how do the cells of imbibed seeds discriminate between stored mRNAs to be utilized in germination and those to be destroyed?
(vi) Do differences exist among stored-mRNAs in terms of the rate of degradation?
(vii) The future challenge, therefore, will be to understand how these multiple noted components are integrated.

## Data Availability

Not applicable.
